# Breast Cancer with Increased Drug Resistance, Invasion Ability, and Cancer Stem Cell Properties through Metabolism Reprogramming

**DOI:** 10.3390/ijms232112875

**Published:** 2022-10-25

**Authors:** Kian-Hwee Chong, Yao-Jen Chang, Wei-Hsin Hsu, Ya-Ting Tu, Yi-Ru Chen, Ming-Cheng Lee, Kuo-Wang Tsai

**Affiliations:** 1 Department of Surgery, Taipei Tzu Chi Hospital, Buddhist Tzu Chi Medical Foundation, New Taipei City 23142, Taiwan; 2 Department of Surgery, School of Medicine, Buddhist Tzu Chi University, Hualien 97004, Taiwan; 3 Department of Research, Taipei Tzu Chi Hospital, Buddhist Tzu Chi Medical Foundation, No. 289 Jianguo Road, Xindian District, New Taipei City 23142, Taiwan

**Keywords:** breast cancer, drug-resistant, metabolism, glycolysis, tricarboxylic acid cycle, hexamine biosynthesis pathway

## Abstract

Breast cancer is a heterogeneous disease, and the survival rate of patients with breast cancer strongly depends on their stage and clinicopathological features. Chemoradiation therapy is commonly employed to improve the survivability of patients with advanced breast cancer. However, the treatment process is often accompanied by the development of drug resistance, which eventually leads to treatment failure. Metabolism reprogramming has been recognized as a mechanism of breast cancer resistance. In this study, we established a doxorubicin-resistant MCF-7 (MCF-7-D500) cell line through a series of long-term doxorubicin in vitro treatments. Our data revealed that MCF-7-D500 cells exhibited increased multiple-drug resistance, cancer stemness, and invasiveness compared with parental cells. We analyzed the metabolic profiles of MCF-7 and MCF-7-D500 cells through liquid chromatography–mass spectrometry. We observed significant changes in 25 metabolites, of which, 21 exhibited increased levels (>1.5-fold change and *p* < 0.05) and 4 exhibited decreased levels (<0.75-fold change and *p* < 0.05) in MCF-7 cells with doxorubicin resistance. These results suggest the involvement of metabolism reprogramming in the development of drug resistance in breast cancer, especially the activation of glycolysis, the tricarboxylic acid (TCA) cycle, and the hexamine biosynthesis pathway (HBP). Furthermore, most of the enzymes involved in glycolysis, the HBP, and the TCA cycle were upregulated in MCF-7-D500 cells and contributed to the poor prognosis of patients with breast cancer. Our findings provide new insights into the regulation of drug resistance in breast cancer, and these drug resistance-related metabolic pathways can serve as targets for the treatment of chemoresistance in breast cancer.

## 1. Introduction

Breast cancer is the most common cancer among women. According to global cancer statistics, approximately 2.3 million new cancer cases were diagnosed in 2020, of which, 11.7% were breast cancer cases, and approximately 42,000 women died of breast cancer in the same year [1,2]. Currently, various public health policies and new clinical treatments have resulted in improved mortality and survival rates [1,3,4]. In particular, breast cancer prevention, routine screening, and precise biomarker development for early detection have all contributed to the improvement in the survival rate of patients with breast cancer. In addition, the development of new treatments, such as anti-HER2 therapy, anti-ER therapy, anti-PI3K and anti-mTOR therapy, and anti-PD1 immunotherapy, have improved the survival rate of patients with breast cancer [5]. So far, chemotherapy remains an effective treatment for breast cancer. Chemotherapeutic drugs mainly inhibit the cell cycle and cancer cell proliferation. However, during chemotherapy, patients develop various side effects, which cause a delay in the timing of the treatment and prolong the duration of treatment. Some patients even stop treatment during the treatment course. All these factors provide an opportunity for breast cancer cells to develop resistance to chemotherapeutic agents [6,7]. The development of resistance to chemotherapy in cancer cells is a key factor in shortening the survival of patients with breast cancer.

Previous studies have elucidated the mechanisms through which cancer cells develop resistance to different therapeutic agents [8]. Intrinsic variety plays crucial roles in determining the response to chemotherapy. Breast cancer cells develop chemoresistance through several mechanisms, including genetic variations, epigenetic modifications, and metabolic pathway alterations [9,10]. In addition to increasing the drug resistance of cancer cells, these molecular mechanisms often result in the enhancement of cancer cell growth and metastatic capacity and lead to the development of cancer stem cell properties [11,12].

The study of the metabolic characteristics of tumors and their relationship with cancer progression is a growing area of cancer research [10,13]. Mitochondrial energy reprogramming in tumors has been identified as a characteristic that accounts for the metabolic plasticity in tumors beyond glycolytic phenotypes [14,15,16]. Furthermore, tumor metabolism regulation is a promising therapeutic strategy. Substantial differences in glucose metabolism can be observed between drug-sensitive and drug-resistant breast cancer phenotypes [17]. In the presence of oxygen, cancer cells mainly obtain energy through aerobic glycolysis, which promotes proliferation and progression as well as resistance to apoptosis [18,19]. Tumor glucose metabolism and glycolysis rates are associated with intrinsic or acquired resistance to routinely used anticancer agents [19,20,21].

Doxorubicin (adriamycin) is a commonly used chemotherapeutic drug, and it is widely employed for the treatment of invasive breast cancer [22]. Clinically, the treatment of patients with breast cancer with doxorubicin for a short period usually causes cancer cells to develop drug resistance and leads to recurrence and treatment failure. To date, many studies have explored the molecular mechanisms underlying doxorubicin resistance, and extensive genetic and molecular analyses have revealed substantial changes in various biological signaling pathways or gene expression, including ABC membrane transporter dysfunction, cell apoptosis-related protein inhibition, aberrant cell cycle-related protein expression, metabolism reprogramming, and signaling transduction pathway alteration (e.g., those in MAPK, PI3K/Akt, RohA/ROCK, Wnt3B, TGF-β, EGFR, and Notch pathways) [23,24,25,26]. Chemotherapy-derived aberrant glucose metabolism is a major type of metabolism reprogramming in breast cancer cells [27]. In addition, chemotherapy can create a pseudo-hypoxic condition, causing cancer cells to considerably depend on the oxidative phosphorylation system (OXPHOS) in mitochondria instead of exhibiting the Warburg effect [28]. Therefore, metabolism reprogramming has been recognized as a mechanism of breast cancer cells, through which they respond to chemotherapy. However, the role of glucose metabolism reprogramming in conferring drug resistance in cancer cells needs to be investigated. To elucidate the possible mechanisms of breast cancer resistance, we analyzed cellular metabolites underlying drug resistance. We believe that metabolic enzymes are potential therapeutic targets for alleviating resistance to chemotherapeutic drugs.

## 2. Results

### 2.1. Generation and Characterization of MCF-7 Cells Resistant to Doxorubicin

**Figure 1:**
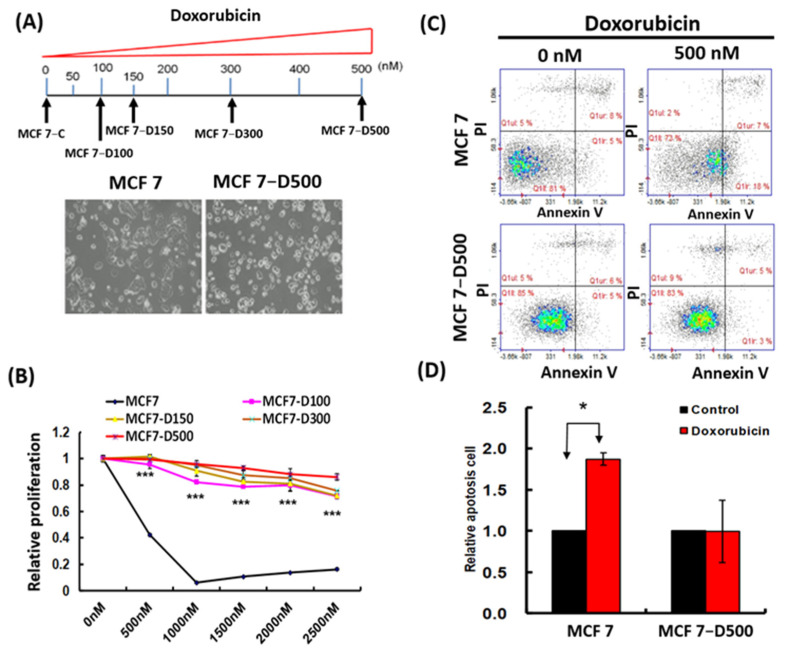

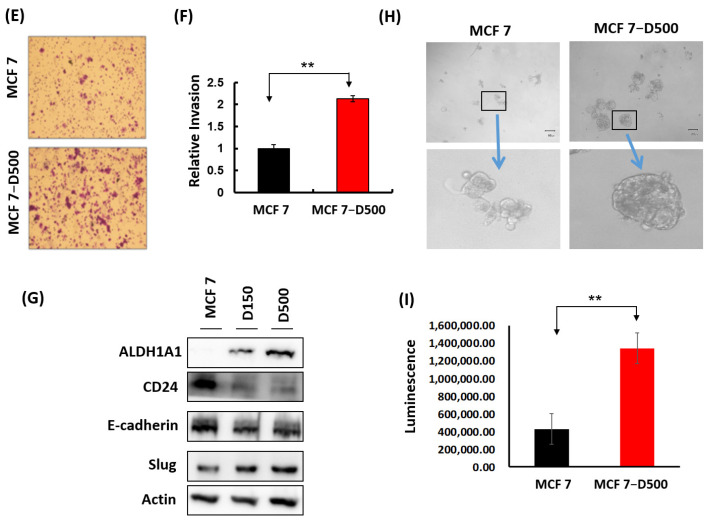
Generation and characterization of MCF-7 cells with doxorubicin resistance. (**A**) The flowchart for establishing doxorubicin resistance in MCF-7 cells and the morphology of MCF-7 control and MCF-7-D500 cells was observed using a microscope. (**B**) The proliferation of MCF-7 parental and doxorubicin-resistant MCF-7 cells (MCF-7-D100, MCF-7-D150, MCF-7-D300, and MCF-7-D500). (**C**,**D**) The apoptosis assay of MCF-7 and MCF-7-D500 cells after doxorubicin (500 nM) treatment for 24 h. (**E**,**F**) The cell motility of MCF-7 and MCF-7-D500 cells. (**G**) The cancer stem cell-related and EMT-related markers were examined. (**H**,**I**) The quantification of the mammosphere-forming efficiency of MCF-7 and MCF-7-D500 cells (* *p* < 0.05; ** *p* < 0.01, *** *p* < 0.001).

**Figure 2:**
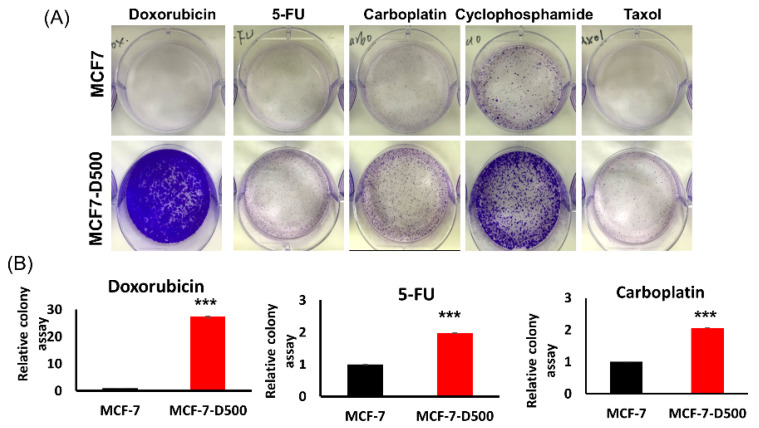

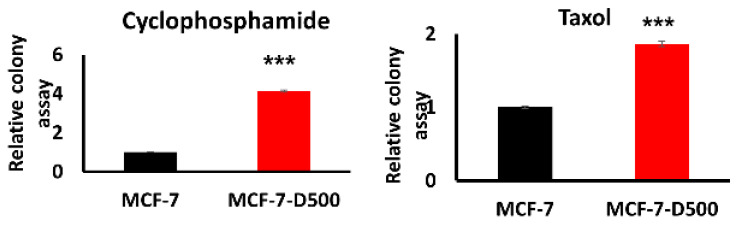
MCF-7-D500 cells with multidrug resistance. (**A**) The cell colony formation ability of MCF-7 and MCF-7-D500 cells was examined after treatment with various chemotherapeutic drugs, including doxorubicin, 5-FU, carboplatin, cyclophosphamide, and taxol. (**B**) The colony formation ability was quantified (*** *p* < 0.001).

To establish a doxorubicin-resistant subline, the MCF-7 cells were treated with a low concentration of doxorubicin (50 nM), and the stepwise selection method was adopted (Figure 1A). Finally, we successfully established a series of drug-resistant cells, namely MCF-7-D100, MCF-7-D150, MCF-7-D300, and MCF-7-D500. Our selection approach mimicked clinical chemotherapy for the formation of minimal residual disease from heterogeneous breast cancer cells. We characterized the drug resistance phenotype of the breast cancer cells and observed that the doxorubicin-resistant cells (MCF-7-D100, MCF-7-D150, MCF-7-D300, and MCF-7-D500) were more resistant to doxorubicin-induced growth inhibition compared with the MCF-7 control cells when treated with various concentrations of doxorubicin (Figure 1B). Furthermore, the MCF-7-D500 cells exhibited resistance to doxorubicin-induced apoptosis after they were treated with 500 nM doxorubicin for 24 h (Figure 1C,D). A breast cancer tumor is highly heterogeneous, which leads to the development of a rare subpopulation of stem-like cells with an inherent predisposition toward drug resistance. Our data revealed that the invasion ability of the MCF-7-D500 cells was significantly increased compared with that of the MCF-7 control cells (Figure 1E,F). In addition, the expression levels of E-cadherin and CD24 were decreased and those of ALDH1A1 and Slug were increased in the MCF-7-D500 cells compared with those in the MCF-7 control cells (Figure 1G and Figure S1). In 3D cultures, the MCF-7-D500 cells demonstrated a greater capacity to form spheroids than did the MCF-7 parental cells (Figure 1H,I). Furthermore, we examined cell viability after treatment with various chemotherapeutic drugs and observed that the MCF-7-D500 cells were more resistant than the MCF-7 cells to 5-fluorouracil, taxol, carboplatin, and cyclophosphamide (Figure 2). Taken together, our data indicate that the MCF-7-D500 cells exhibited higher multidrug resistance, stemness, and invasion ability than did the MCF-7 control cells.

### 2.2. Metabolism Reprogramming Involved in the Drug Resistance of Breast Cancer Cells

**Figure 3:**
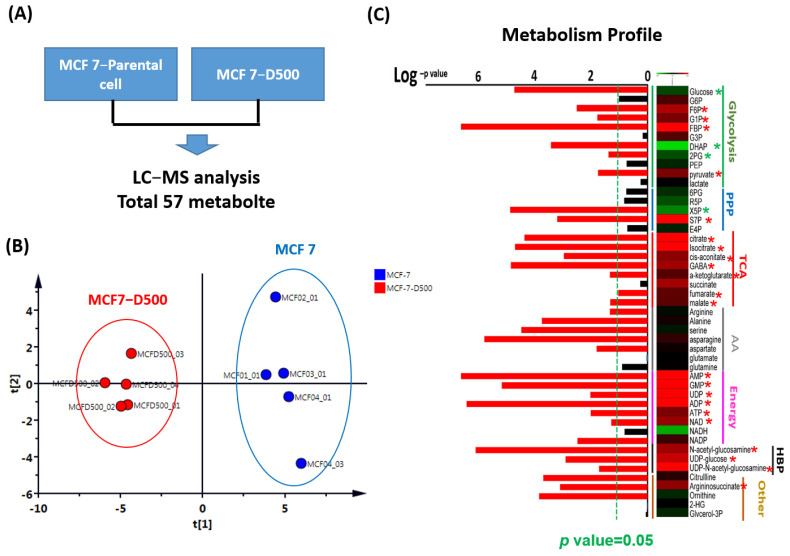
Metabolic profiles of MCF-7 cells with drug resistance examined through LC–MS. (**A**) The flowchart to identify the differential expression of metabolic products. (**B**) Principal coordinate analysis based on the number of metabolic products in all samples. (**C**) The heatmap of the selected principal component analysis from targeted metabolites in cells. The green dotted indicates *p* < 0.05 (* *p* < 0.05).

To explore the contribution of metabolism to drug resistance, we examined the metabolic profiles of the MCF-7 cells with or without doxorubicin resistance through LC-MS (Figure 3A). The results of the principal coordinate analysis revealed that the distribution of metabolic products was similar among the five replicate samples (Figure 3B). A total of 47 metabolic products were detected in our samples, of which 21 exhibited significantly increased levels (>1.5-fold and *p* < 0.05) and 4 exhibited significantly decreased levels (<0.75-fold and *p* < 0.05) in the MCF-7 cells with doxorubicin resistance (Figure 3C).

### 2.3. Glycolysis-Associated Enzymes Promoted Glucose Consumption

**Figure 4:**
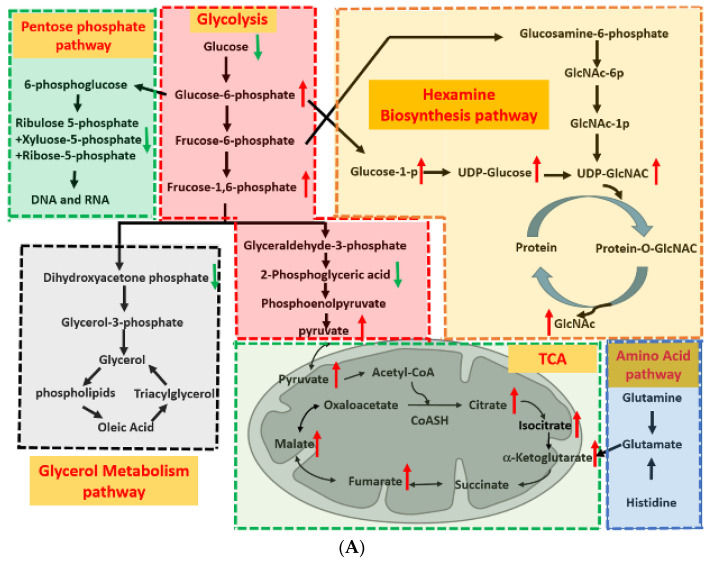

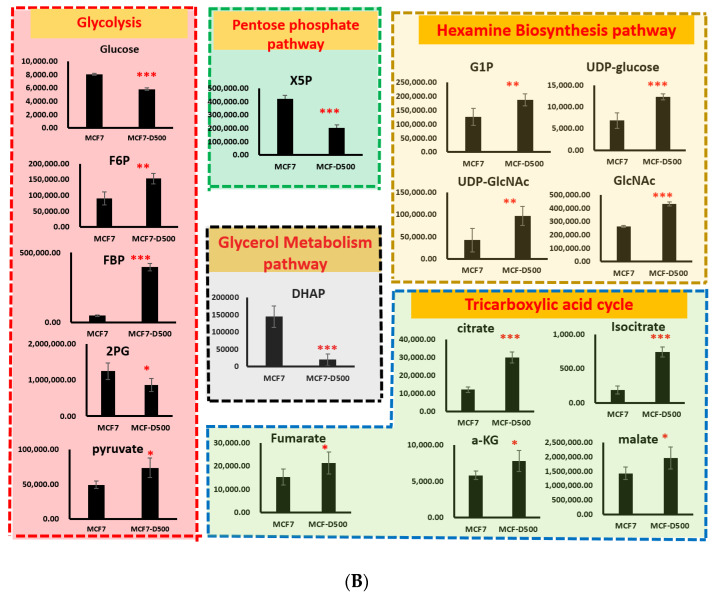
Schematic of metabolic pathways involved in MCF-7 cells with drug resistance. (**A**) Overview of changes in metabolic products in integrated energy metabolism pathways. The green frame indicates the pentose phosphate pathway. The red frame indicates the glycolysis pathway. The gray frame indicates the glycerol metabolism pathway. The green frame indicates the TCA cycle. The yellow frame indicates the hexamine biosynthesis pathway, and the blue frame indicates the amino acid metabolic pathway. Red arrows indicate metabolic products upregulated in the MCF-7-D500 cells, and green arrows indicate downregulated metabolic products. (**B**) The expression levels of metabolites significantly differed between the MCF-7 and MCF-7-D500 cells in their respective pathways. (* *p* < 0.05; ** *p* < 0.01, *** *p* < 0.001).

Because energy is crucial to maintain cancer cell growth, the altered equilibrium concentrations of energy metabolites may indicate impaired cellular physiology. Our metabolic profiles revealed that several energy-related metabolite levels, including AMP, GMP, UDP, ADP, ATP, and NAD+, were significantly increased in the MCF-7 cells with drug resistance (Figure 3C). The activation of energy-producing pathways indicates that cells are continually activating various metabolic pathways. As presented in Figure 4, the concentrations of one or more metabolites in five pathways were significantly altered in the breast cancer cells with drug resistance (Figure 4A,B). In particular, glycolysis, the TCA cycle, and the HBP exhibited considerable activity in the drug-resistant cells compared with the parental cells (Figure 4A,B). These results strongly indicate that metabolism reprogramming might be involved in breast cancer drug resistance, including the activation of glycolysis, the TCA cycle, the HBP, and energy production.

### 2.4. Glycolysis and TCA Cycle Were More Active in Drug-Resistant Cells

The glucose concentration was significantly decreased in the drug-resistant cancer cells compared with the parental cells (0.75-fold change, *p* < 0.001; Figure 4A,B), implying that glucose is rapidly consumed in drug-resistant cells. However, the levels of fructose 6-phosphate, fructose 1,6-bisphosphate, and pyruvate increased by 1.7-, 1.5-, and 1.5-fold (*p* = 0.003, *p* < 0.001, and *p* = 0.02, respectively), whereas the level of 2-phosphoglyceric acid decreased by 0.70-fold (*p* = 0.04). In addition, we observed that the levels of citrate, isocitrate, a-ketoglutarate, fumarate, and malate increased by 2.5-, 3.9-, 1.5-, 1.7-, and 1.5-fold in the drug-resistant cells compared with the parental cells (*p* < 0.001, *p* < 0.001, *p* < 0.001, *p* = 0.05, *p* = 0.05, and *p* = 0.05, respectively). These results suggest that glycolysis and the TCA cycle may be more active in drug-resistant cells than in normal cells and thus produce more energy to support the proliferation and metastasis of cancer cells.

### 2.5. HBP Activity in Drug-Resistant Cells

Although most glucose is primarily metabolized through glycolysis for energy production, a small amount is shunted to the pentose phosphate pathway (PPP) and the HBP through the accumulation of fructose 6-phosphate and glucose 6-phosphate (G6P). Our data indicated that the levels of glucose-1-phosphate, UDP-glucose, UDP-N-acetyl-glucosamine, and N-acetyl-glucosamine significantly increased by 1.6-, 1.8-, 2.3-, and 1.6-fold, respectively, in the drug-resistant cells (*p* = 0.02, *p* = 0.001, *p* = 0.02, and *p* < 0.001, respectively; Figure 4A,B). The high HBP-related metabolite concentrations suggest that accumulated G6P is shunted from the glycolytic pathway to the HBP, resulting in the HBP pathway being more active in drug-resistant cells.

### 2.6. Metabolism-Related Gene Expression in Breast Cancer Cells with Drug Resistance

**Figure 5:**
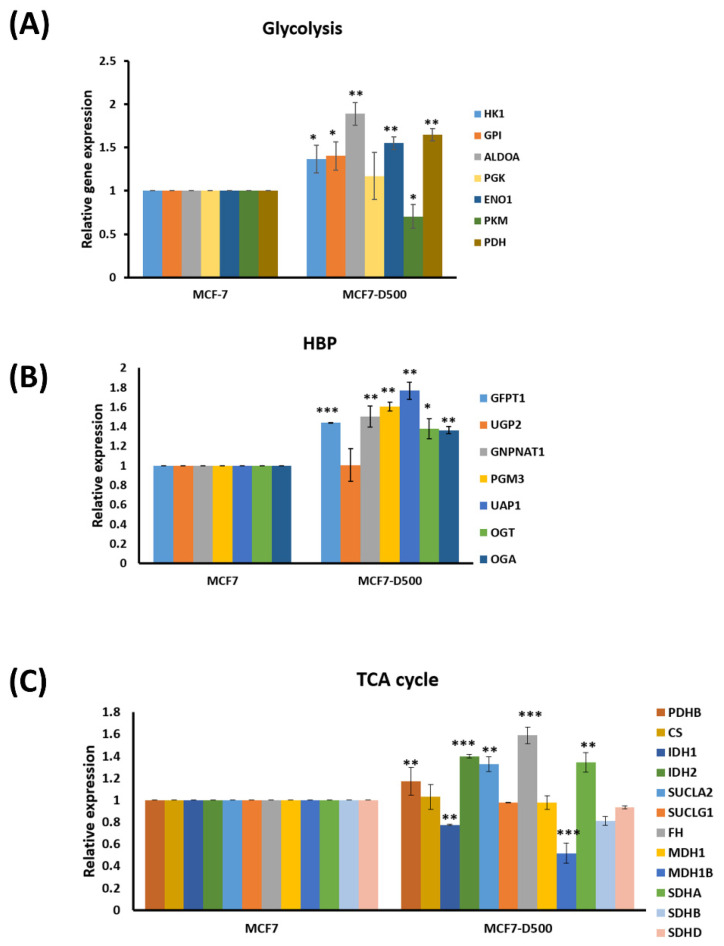
Expression levels of metabolic enzymes were examined in MCF-7 and MCF-7-D500 cells through real-time PCR: (**A**) Glycolysis-related genes; (**B**) HBP-related genes, and (**C**) TCA cycle-related genes. All real-time PCR experiments were performed in triplicate (* *p* < 0.05; ** *p* < 0.01, *** *p* < 0.001).

The metabolic products of glycolysis, the TCA cycle, and the HBP were increased in the MCF-7-D500 cells compared to the MCF-7 parental cells, implying that these pathways were constitutively activated in the MCF-7 cells with drug resistance. Therefore, we speculated that the hyperactivation of these pathways results from the abnormal expression of metabolic enzymes in drug-resistant cells. The findings of quantitative PCR revealed that the expression levels of most of the genes related to glycolysis (*HK1*, *GPI*, *ALDOA*, *ENO1*, and *PDH1*; Figure 5A), the HBP (*GFPT1*, *GNPNAT1*, *PGM3*, *UAP1*, *OGT*, and *OGA*; Figure 5B), and the TCA cycle (*PDHB*, *IDH2*, *SUCLA2*, *FH*, and *SDHA*; Figure 5C) were significantly increased in the MCF-D500 cells compared with the MCF-7 cells. These results suggest that the high expression of glycolysis-related genes results in rapid glucose consumption, leading to the production of high amounts of metabolites related to the HBP and TCA in the MCF-7 drug-resistant cells.

**Figure 6:**
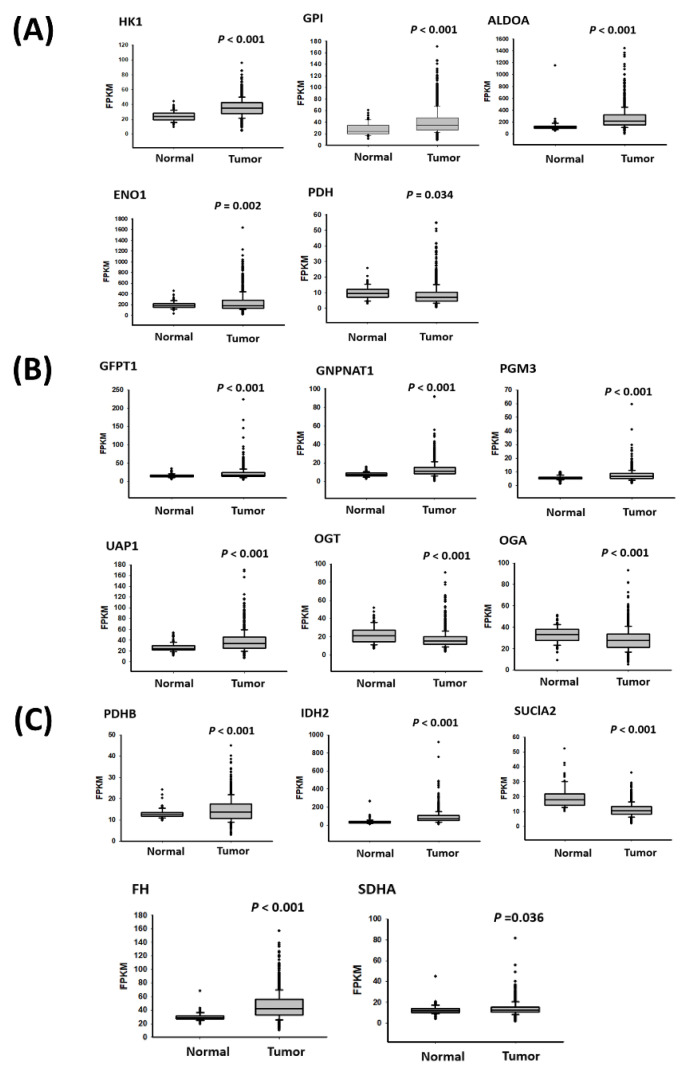
Expression levels of (**A**) glycolysis-associated enzymes, (**B**) HBP-related enzymes, and (**C**) TCA cycle-related enzymes were examined in breast cancer by analyzing data from the TCGA database.

**Figure 7:**
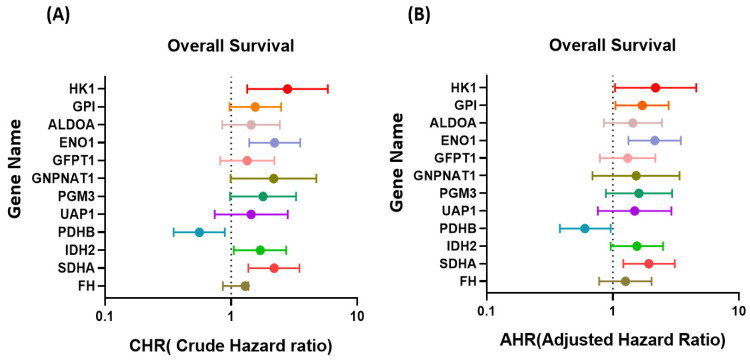
Correlation between overall survival and glycolysis-, HBP-, and TCA cycle-related enzymes was examined using data from the TCGA database. (**A**) CHR (crude hazard ratio) and (**B**) AHR (adjusted hazard ratio). The filled circle indicated the Crude or Adjusted hazard ratios of individual genes.

We further examined the expression levels and clinical impacts of metabolic enzymes in breast cancer by using the TCGA database. Among the metabolic enzymes with abnormal expression, the expression levels of the genes related to glycolysis (*HK1*, *GPI*, *ALDOA*, and *ENO1*; Figure 6A), the HPB (*GFPT1*, *GNPNAT1*, *PGM3*, and *UAP1*; Figure 6B), and the TCA cycle (*PDHB*, *IDH2*, *FH*, and *SDHA*; Figure 6C) were significantly increased in the breast cancer tissues compared to the corresponding adjacent normal tissues. Furthermore, we examined the clinical impact of these metabolic enzymes by using TCGA (Tables S3–S9). As presented in Tables S3–S8, the high expression of GPI and FH was significantly associated with the pT stage (*p* = 0.003 and *p* = 0.005, respectively), and the high expression of GNPNAT1 was significantly associated with the pN stage (*p* = 0.01). In addition, the high expression of SDHA was associated with the poor pathological stage (*p* = 0.002), pT stage (*p* = 0.005), and pM stage (*p* = 0.025). We investigated the effect of the expression of these genes on the overall survival of the patients with breast cancer, and the results revealed that the high expression levels of *HK1* (crude hazard ratio [CHR] = 2.80; 95% CI = 1.34–5.84, *p* = 0.006), *ENO1* (CHR = 2.21; 95% CI = 1.39–3.52, *p* = 0.001), *IDH2* (CHR = 1.7; 95% CI = 1.05–2.73, *p* = 0.029)*, PDHB* (CHR = 0.56; 95% CI = 0.35–0.89, *p* = 0.015), and *SDHA* (CHR = 1.70; 95% CI = 1.05–2.73, *p* = 0.029) were significantly associated with shorter overall survival in the patients with breast cancer (Table S9 and Figure 7A). The results of the multivariate analysis indicated that the high expression levels of six metabolic genes, *HK1* (adjusted hazard ratio [AHR] = 2.18; 95% CI = 1.04–4.59, *p* = 0.04), *GPI* (AHR = 1.71; 95% CI = 1.05–2.77, *p* = 0.03), *ENO1* (AHR = 2.15; 95% CI = 1.33–3.46, *p* = 0.002), *PDHB* (AHR = 0.6; 95% CI = 0.38–0.96, *p* = 0.034), and *SDHA* (AHR = 1.93; 95% CI = 1.21–3.09, *p* = 0.006), were independent prognostic biomarkers for overall survival (Table S9 and Figure 7B).

Taken together, our data indicated that breast cancer cells with drug resistance develop enhanced invasion ability through metabolism reprogramming, especially that of glycolysis, the HBP, and OXPHOS dependence in chemotherapy-resistant breast cancer. Our findings indicate that drug resistance-related metabolic pathways can serve as targets for the treatment of chemoresistance in breast cancer.

## 3. Discussion

Despite the development of many clinical treatments for patients with breast cancer, including surgery, radiation therapy, chemotherapy, immunotherapy, and targeted therapy, to date, drug-resistant cells lead to tumor recurrence and metastasis, which are the main causes of cancer-related mortality in patients with advanced breast cancer [29]. The Warburg effect discovered by Otto Warburg indicates that cancer cells mostly utilize glycolysis and consume a high amount of glucose [30]. Furthermore, the Warburg effect can increase the rate of glucose uptake to produce ATP through anaerobic glycolysis and thus accelerate cell proliferation and cancer progression as well as increase drug resistance [31]. Therefore, reprogrammed metabolism is a crucial hallmark of breast cancer that promotes cancer cell growth and metastasis and induces drug resistance [19,32,33]. However, upregulated OXPHOS and heterogeneity in tumor metabolism have been observed in many types of cancer [34,35]. Tumor metabolism mainly involves altered glucose metabolism, which plays roles in driving cancer progression, determining the response to cancer treatment, and demonstrating resistance to treatment. Lyon et al. reported that glycolysis is critical for ATP production in drug-resistant breast cancer cells compared with drug-sensitive breast cancer cells, suggesting that metabolites contribute to the development of drug resistance [17]. The results of this study revealed high glucose consumption in the MCF-7 cells with drug resistance. Similarly, Ahmadpour et al. demonstrated that lactate production and glucose consumption were higher in MCF-7 cells with doxorubicin resistance than in parental MCF-7 cells, suggesting that MCF-7 drug-resistant cells exhibit a predominance of glycolytic metabolism [36]. Our data revealed that in the MCF-7-D500 cells, the G6P and FBP levels were significantly increased, whereas those of 2PE and DHAP were significantly decreased. In previous studies, the lactate level was increased in breast cancer cells treated with cisplatin or doxorubicin, suggesting enhanced glycolytic activity in breast cancer cells after chemotherapy [37,38]. Because lactate is the end product of glycolysis, its accumulation implies increased anaerobic glycolysis. However, in the present study, we did not observe changes in the lactate level in the MCF-7 cells with doxorubicin resistance compared with the parental MCF-7 cells. Previous studies revealed that the lactate level significantly increased only after the treatment of MCF-7 or MBA-MB-231 cells with doxorubicin [37,38]. Therefore, we suggested that metabolic pathways may be reprogrammed from anaerobic respiration to aerobic respiration (the HBP and TCA cycle) during breast cancer cells developing drug resistance.

A high accumulation of glucose-6-phosphate dehydrogenase (G6PD) might cause G6PD to be shunted from glycolysis to the PPP or HBP. Furthermore, the G6PD expression level was elevated in the advanced cancer stage or metastatic disease, which was associated with poor treatment outcomes [39,40]. Min et al. reported that G6PD expression along with the upregulation and metabolites of 3-phosphoglycerate and ribulose-5-phosphate production were increased in paclitaxel-resistant cells [41]. In addition, Luo et al. demonstrated that G6PD plays a crucial antioxidant regulator role in the metastasis and drug resistance of breast cancer cells [42]. However, we observed a contrasting finding; the expression levels of PPP metabolites did not change, but the level of xylulose-5-phosphate decreased in the doxorubicin-resistant MCF-7 cells. These results imply that the PPP might not be involved in the development of doxorubicin resistance in MCF-7 cells.

Reactive oxygen species (ROS) production in mitochondria was higher in MCF-7 cells with doxorubicin resistance than in parental MCF-7 cells [36]. Excessive ROS production may lead to a glycolysis reaction switch to the HBP through the inhibition of the glycolytic enzyme GAPDH [43,44]. In addition, the inhibition of GAPDH activity causes a decrease in its downstream metabolites. Therefore, we observed that the 2-Phosphoglyceric acid (2-PG) level was significantly decreased in the MCF-7 cells with doxorubicin resistance (Figure 3 and Figure 4). Fructose-6-phosphate amidotransferase (GFAT1) is a rate-limiting enzyme of the HBP. The expression level of GFAT1 was significantly increased in human cancer cells [45]. O-GlcNAcylation is a nutrient sensor that is sensitive to metabolic states, including glucose, amino acids, fatty acids, and nucleotides. Therefore, the abnormal hyper- or hypo-O-GlcNAcylation of cellular proteins contributes to severe systemic disorders, including cancer [46]. The O-GlcNAcylated proteins increased after short-term treatment with doxorubicin, suggesting that abnormalities in O-GlcNAcylation occur during chemotherapy [47]. Liu et al. reported that the HBP plays a key role in the cellular metabolic response to chemotherapy by regulating O-GlcNAcylation, and this has crucial implications for drug resistance [47]. They demonstrated that the high level of O-GlcNAcylation was a key factor in drug resistance but was not associated with *OGT* or *OGA* gene expression [47]. Liu et al. reported that the GFAT protein level significantly increased during doxorubicin treatment [47]. This finding implies that the glycolysis pathway might be shunted into the HBP after doxorubicin treatment. In this study, we observed that the MCF-7 cells with doxorubicin resistance accumulated higher amounts of glucose-1-p, UDP-glucose, and UDP-GlcNAc than did the parental MCF-7 cells. In particular, UDP-GlcNAc is the end product of the HBP and can be a substrate for protein glycosylation, implying that the O-GlcNAcylation of proteins might be increased in the MCF-7 cells with drug resistance in this study. Therefore, the inhibition of O-GlcNAcylation can be a useful strategy for preventing doxorubicin resistance in breast cancer. The combination of O-GlcNAcylation inhibitors and chemotherapy may be a new strategy for cancer treatment.

For tumor proliferation, survival, metastasis, and drug resistance, mitochondrial metabolism is crucial. Furthermore, cancer cell resistance to chemotherapy and radiotherapy is associated with mitochondrial OXPHOS dependence. A study indicated that drug-resistant cancer cells are dependent on mitochondrial OXPHOS for survival. The inhibition of OXPHOS with complex I inhibitors prolonged survival in mouse xenograft models [28]. Similarly, in our study, we observed that the TCA cycle demonstrated high activity in the MCF-7 cells with doxorubicin resistance. These findings suggest that MCF-7 cells with doxorubicin resistance might shift from anaerobic metabolism to oxidative phosphorylation in response to increased energy needs. Echeverria et al. reported that OXPHOS activation significantly increased in residual breast tumors following chemotherapy [48]. This finding is consistent with that of our study that metabolism reprogramming can alleviate doxorubicin-induced damage and promote cancer cell growth and metastasis. Therefore, the development of an inhibitor to counteract the activation of OXPHOS and the TCA cycle can be a new approach to the treatment of drug resistance and tumor metastasis. In acute myeloid leukemia cells resistant to cytarabine, OXPHOS activation was elevated, and targeting mitochondrial metabolism alleviated tumor resistance to cytarabine [49]. Evans et al. performed an RNA transcriptome analysis of patients with triple-negative breast cancer (TNBC) who received neoadjuvant chemotherapy and observed that high OXPHOS activation was associated with poor outcomes [15]. Treatment with an OXPHOS inhibitor (IACS-01759) completely destroyed xenografts obtained from patients with chemotherapy-resistant TNBC. Moreover, complete regression was observed in a patient-derived xenograft model (PDX) after IACS-01759 treatment [15]. In addition, the expression levels of MYC and LCL1 increased in chemotherapy-resistant TNBC cells, resulting in enhanced OXPHOS activation and ROS production [50].

## 4. Material and Methods

### 4.1. Cell Line and Doxorubicin Resistance of Breast Cancer Cells

The human breast cancer cell line MCF-7 was obtained from the American Type Culture Collection and was maintained in Dulbecco’s modified Eagle’s medium supplemented with 10% inactivated fetal bovine serum (Invitrogen, Carlsbad, CA, USA). To establish a doxorubicin-resistant subline, various concentrations of doxorubicin were used to induce resistance in MCF-7 cells by adopting the stepwise selection method. MCF-7 cells were grown in cell culture medium containing a doxorubicin concentration of 50 nM. When the cells reached an appropriate confluence at a certain concentration, they were passaged, and double the previous doxorubicin concentration was used for the stepwise selection of resistant cells; a final doxorubicin concentration of 500 nM was applied. Finally, we obtained a series of subline cells with resistance to various dosages of doxorubicin (MCF-7-D100, MCF-7-D150, MCF-7-D300, and MCF-7-D500), and parental MCF-7 cells were used as the control.

### 4.2. Cell Proliferation and Migration Assay

To examine the proliferation of breast cancer cells, 5000 living cells were plated on 96-well plates. Cell growth was determined on 0, 1, 2, 3, and 4 days by using the 3-(4,5-dimethylthiazol-2-yl)-2,5-diphenyltetrazolium bromide assay (MTT assay; Sigma, Billerica, MA, USA). The migration ability of the cells was tested in vitro by using a transwell chamber (Corning Costar, Lowell, MA, USA). First, the cells were added to the upper chamber of a transwell with a polycarbonate membrane (containing 8 µm pores). After incubation for 24 h at 37 °C, the cells migrated to the lower side and were detected through Giemsa staining. The number of the migrated cells was determined using a microscope at 200× magnification. All the experiments were repeated 3 times.

### 4.3. Colony Formation Assay

A total of 2000 cells were seeded into each well of a 6-well plate, and the cells were transfected with various concentrations of chemotherapeutic drugs or control in the 6-well plate. The cells were incubated at 37 °C for 10 days. Then, colonies were fixed with 4% formaldehyde for 2 min and stained with crystal violet solution (containing 0.5% crystal violet, 5% formaldehyde, 50% ethanol, and 0.85% sodium chloride) for 2 h. The wells were rinsed with H_2_O after air drying. The crystal violet staining of the cells in each well was solubilized using 1 mL of 10% acetic acid per well, and the absorbance (optical density) of the solution was measured using a spectrophotometer at a wavelength of 595 nm.

### 4.4. RNA Extraction and Real-Time Polymerase Chain Reaction

Total RNA of the MCF-7 and MCF-7-D500 cells was extracted using the EasyPrep total RNA kit (*Biotools*, Taipei, Taiwan). The concentration, purity, and amount of total RNA were determined using the Nanodrop 1000 spectrophotometer (Nanodrop Technologies Inc., Wilmington, DE, USA). Subsequently, 2 µg of the total RNA were reverse-transcribed into cDNA by using oligo (dT)15 primers and the ToolScript MMLV RT kit (*Biotools*, Taipei, Taiwan). Briefly, reverse transcription was performed at 42 °C for 1 h. Finally, the reaction was stopped by the addition of inactivated reverse transcriptase at 85℃ for 5 min. The cDNA was used for performing real-time polymerase chain reaction (PCR) with gene specific-primers, and gene expression was detected using the SYBR Green I assay (*Biotools*, Taipei, Taiwan). Glyceraldehyde-3-phosphate dehydrogenase (GAPDH) was used as an internal control. The primer sequences are listed in Table S1.

### 4.5. Western Blotting

Total proteins were separated through sodium dodecyl sulfate–polyacrylamide gel electrophoresis in 10% polyacrylamide gel. Then, the separated proteins were transferred onto a nitrocellulose membrane (Millipore, Billerica, MA, USA). A blocking buffer (phosphate-buffered saline [PBS]–Tween containing 5% skim milk) was used to block the membrane at 4℃ overnight. Then, the membrane was incubated with primary antibodies for 1 h in PBS–Tween containing 5% skim milk. Subsequently, the membrane was incubated with anti-rabbit or mouse immunoglobulin G horseradish peroxidase-conjugated secondary antibody (1:10,000, Santa Cruz Biotechnology, Inc., Santa Cruz, CA, USA) for 1 h at room temperature. The membrane was washed 3 times with PBS–Tween, and individual protein expression was detected using an electrochemiluminescence kit (*Biotools*, Taipei, Taiwan). The antibodies are listed in Table S2.

### 4.6. Metabolic Profiling by Liquid Chromatography–Mass Spectrometry

The metabolic profiles of the MCF-7 and MCF-7-D500 cells were examined through ultra-high-performance liquid chromatography coupled with mass spectrometry (MS; Xevo-TQS, Waters, Wilford, MA, USA). MS was performed in the negative and positive ion modes with the multiple reaction monitoring mode. Major tandem MS fragment patterns of each analyte were determined using the tuning method. The optimized parameters were as follows: capillary voltage at 1 kV, desolvation temperature at 500 °C, source temperature at 150 °C, and gas flow at 1000 L/h. Chromatographic separation was achieved on a BEH C18 column (100 × 2.1 mm, particle size: 1.7 μm; Waters Corp.) at 45 °C with eluent A (water with 10 mM tributylamine and 15 mM acetic acid) and eluent B (50% acetonitrile with 10 mM tributylamine and 15 mM acetic acid), and the flow rate was set at 0.4 mL/min. MixQC samples (all the analyzed sample mix) were prepared for analysis during analytical runs after every 10th sample. In this study, each sample was analyzed in 5 replicates.

### 4.7. Expression Data from the Cancer Genome Atlas

The transcriptome expression data and clinical information of breast cancer cells were downloaded from The Cancer Genome Atlas (TCGA) portal (https://tcga-data.nci.nih.gov/tcga/dataAccessMatrix.htm, 29 January 2018). The expression profiles and clinical information of 1092 breast cancer tissues and 113 adjacent normal tissues were obtained from the TCGA portal. In this study, the transcriptome profiles of 1092 patients with breast cancer were used to determine overall survival by using the Kaplan–Meier method. The differential expression of genes related to glycolysis, the hexamine biosynthesis pathway (HBP), and the tricarboxylic cycle in breast cancer was examined. Results with *p* < 0.05 were considered statistically significant.

### 4.8. Statistical Analysis

The levels of metabolic products in the MCF-7-D500 and MCF-7 cells were analyzed through liquid chromatography–mass spectrometry (LC–MS), and the differential gene expression was examined using Student’s *t*-test. Cell proliferation, colony formation, and cell migration experiments were performed in triplicate. Histograms present mean values, and error bars indicate standard deviations. The expression levels of metabolic enzymes were examined through real-time PCR or Student’s *t*-test using TCGA data. For overall survival analysis, we performed a receiver operating characteristic curve analysis to define a cutoff value for the expression of these drug resistance-related metabolic enzymes. The patients with breast cancer were separated into two groups according to the cutoff value: those with high expression levels and those with low expression levels. Finally, cumulative survival curves were plotted using the Kaplan–Meier method, and a comparison between the survival curves was performed using the log-rank test. These data were analyzed using Student’s *t*-test. The difference was considered significant when *p* < 0.05.

## 5. Conclusions

Here, we provide evidence for metabolism reprogramming, especially that of glycolysis, the HBP, and OXPHOS dependence, in chemotherapy-resistant breast cancer. More research is needed to validate these findings. These drug resistance-related metabolic pathways can serve as targets for the treatment of chemoresistance in breast cancer.

## Data Availability

Not applicable.

## Supplementary Materials

The following supporting information can be downloaded at: https://www.mdpi.com/article/10.3390/ijms232112875/s1.
