# Supplementary material for: Breast Cancer with Increased Drug Resistance, Invasion Ability, and Cancer Stem Cell Properties through Metabolism Reprogramming
Source: Int J Mol Sci. 2022 Oct 25;23(21):12875. doi: 10.3390/ijms232112875 (PMC9658063; doi:10.3390/ijms232112875)
Supplement: Supplementary file 1 [file ijms-23-12875-s001.zip › ijms-1978302-supplementary.pdf]

Breast Cancer with Increased Drug Resistance, Invasion Ability, and Cancer Stem Cell Property Through Metabolism Reprogramming

Supplementary Figure and Table

(A)

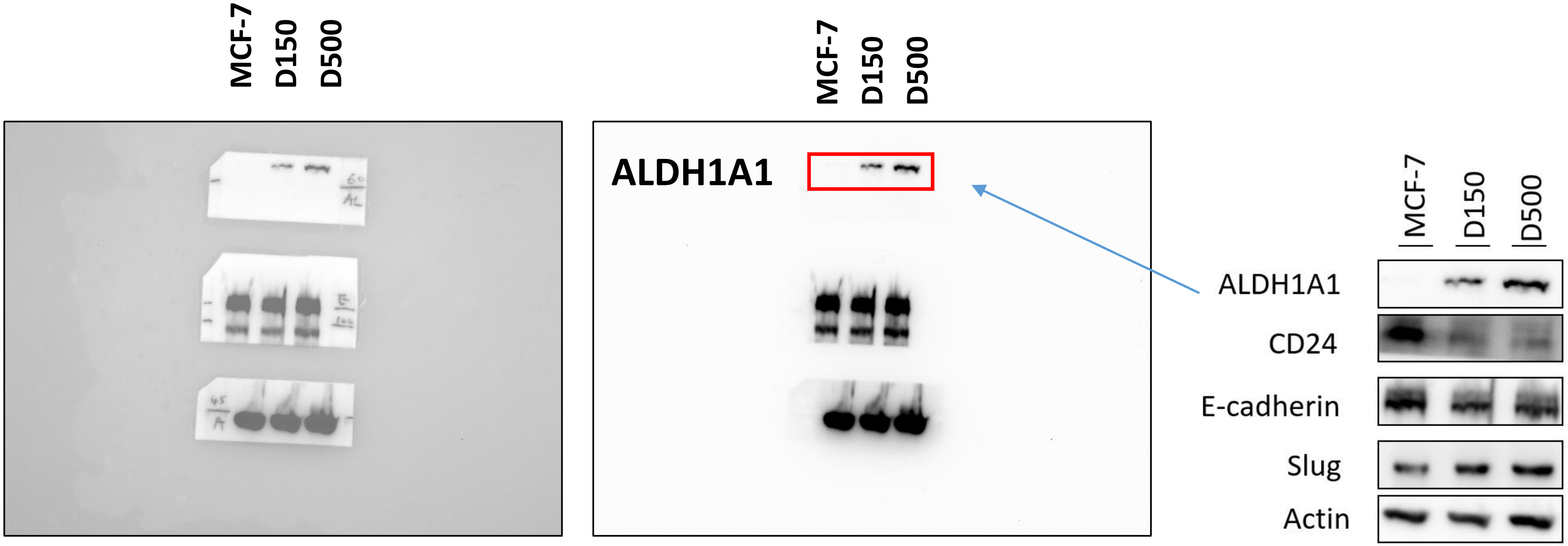

Supplementary Figure S1

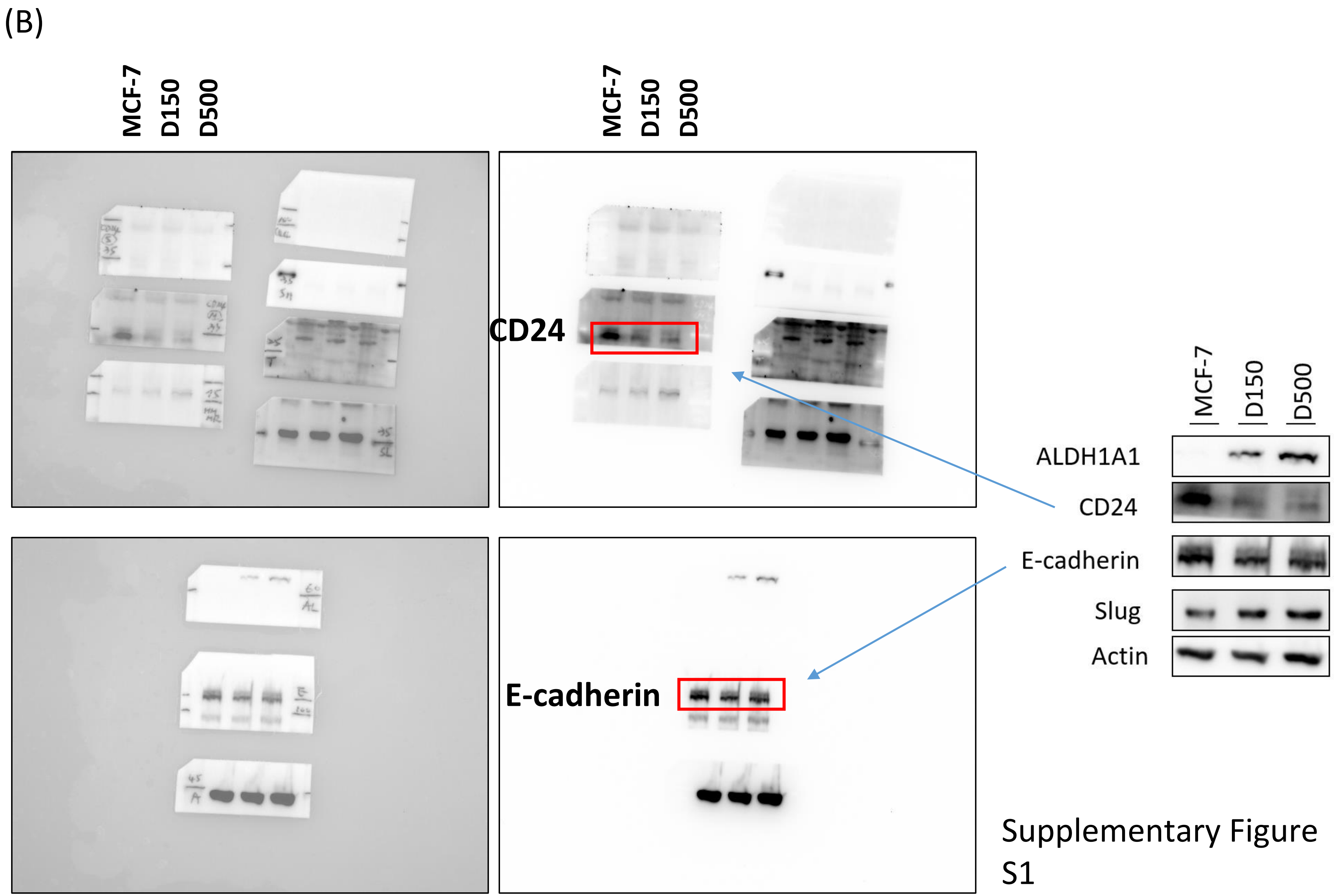

Supplementary Figure S1

(C)

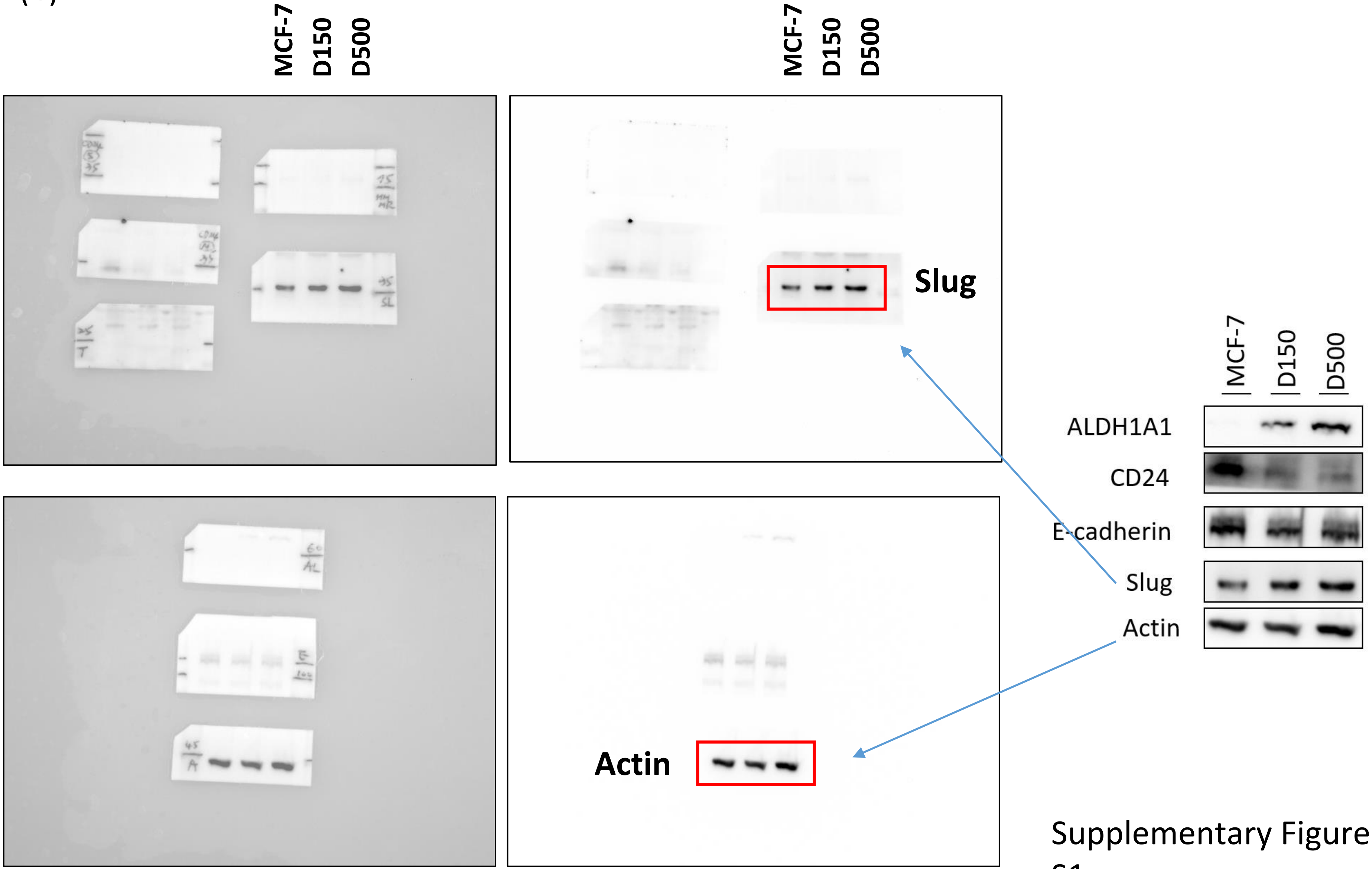

Supplementary Figure S1

Supplementary Table S1. The Primers used in this study of qPCR

**Primer**	**5'~3'**
HK1-F	CGCAGCTCCTGGCCTATTAC
HK1-R	CATGATTCACTTGCACCCGC
PGI-F	CGACTAGTGCACAGGGAGTG
PGI-R	TGGTTGAAGCGGTCCTTGTT
ALDOA-F	AGTCCCGCGTTCTCTCCTTG
ALDOA-R	GGTAGTAGCAAGTTCCTGGCA
PGK-F	CCACTGTGGCTTCTGGCATA
PGK-R	ATGAGAGCTTTGGTTCCCCG
ENO1-F	TTACCACAACCTGAAGAATG
ENO1-R	TCCAGGCCTTCTTTATTCTC
PKM-F	CTTCGTCTTTGCAGCGTAGC
PKM-R	GCTGGGCCAATGGTACAGAT
PDH-F	GGATCCTCCACCCGTCCAAA
PDH-R	TGGGATTCCAATTCGTCTGGG
GFPT1-F	AGATTGCCCACCGAAGCTC
GFPT1-R	TTTTGCAGGCATTGGCTTCC
UGP2-F	GCAGGAGCAAAATGCCATTGACA
UGP2-R	CAGAAAACGGCTCCTTGGCACA
GNPNAT1-F	CAGGGCCTCTACGGACC
GNPNAT1-R	GGCTGACAACTCCAGTCTCTG
PGM3-F	AGGGACCCAGGATAAGGTTGA
PGM3-R	CGTTCGAAATCCAGCAGTCC
UAP1-F	AGCCAGACAAACCCAATGGA
UAP1-R	ATTTGTAGCACTGCGGGGAA
OGT-F	CGAAACGTATGCTTTCCTTCCA
OGT-R	GCAACTGCCCTCTTTCCTTG
OGA-F	GATGTAGTGATGAGTAGGCAAGT
OGA-R	CCTGGCTCATAATGGGCTCC
PDHB-F	CCTTCGGGAGGTGACAGTTC
PDHB-R	ATTCACAAATGGGCCGCAAC
CS-F	CCCCATGTCTCAGCTCAGTG
CS-R	AGTCAATGGCCCCAATACCG
IDH1-F	CTATGATGGTGACGTGCAGTCG
IDH1-R	CCTCTGCTTCTACTGTCTTGCC
IDH2-F	AGATGGCAGTGGTGTCAAGGAG

IDH2-R	CTGGATGGCATACTGGAAGCAG
SUCLA2-F	GCAAGAAGCTGGTGTCTCCGTT
SUCLA2-R	CCACCAGCTAAAACCTGTGCCT
SUCLG1-F	GTTGGAGGAACCACTCCAGG
SUCLG1-R	GGGCCCAATTAGCCTTGTCT
FH-F	ATCATGCCAGGCAAGGTGAA
FH-R	GGAAACTGAAGCATCCCCCA
MDH1-F	CGGTGTCCTAATGGAACTGCAAG
MDH1-R	CATCCAGGTCTTTGAAGGCAACG
MDH1B-F	AGAGCTGTTGGATCGTGGAGGA
MDH1B-R	GTTCAGTCGTCATGCTAGAGGTG
SDHA-F	ACTGTTGCAGCACAGCTAGA
SDHA-R	GGCACTCCCCATTCTCCATC
SDHB-F	GTGGCCCCATGGTATTGGAT
SDHB-R	AGTTGCTCAAATCCTTATTGAGGT
SDHD-F	GAGCTGGCTCCAAGGCTG
SDHD-R	TTTCTGCAAGGCATCCCCAT

Supplementary Table S2. The antibodies used in this study

Antibody name	1st Ab Dilution	MW (kDa)	Company	Host	2nd Ab Dilution
ALDH1A1	1:1000	56	SC-374076, Santa Cruz	Mouse	1:5000
CD24	1:1000	35-70	555426, BD Biosciences	Mouse	1:5000
E-cadherin	1:4000	135	GTX61329, GeneTex	Rabbit	1:5000
E-cadherin	1:1000	30	SC-166476, Santa Cruz	Mouse	1:5000
β-actin (ACTB)	1:5000	43	MAB1501, Millipore	Mouse	1:5000

*MW: Molecular Weight

Supplementary Table S3. Correlation of HK1 and GPI expression with clinicopathological characteristics of breast cancer 1070 patients.

Variables	HK1 (n=1070)				GPI (n=1070)		
	No. (%)	Mean±SD	Median	p-value	Mean±SD	Median	p-value
**Pathology stage**							
I	272 (25.4)	35.29±11.31	35.53	0.826[a]	38.16±18.12	34.37	0.051[b]
II	554 (51.8)	36.21±21.25	34.82		42.79±23.77	35.18	
III	224 (20.9)	35.60±11.97	33.58		39.17±21.89	33.19	
IV	20 (1.9)	38.07±13.24	38.73		49.28±25.21	39.68	
**pT stage**							
T1	355 (33.2)	35.42±11.26	35.41	0.926[a]	37.64±18.55[de]	33.33	0.003[b]
T2	560 (52.3)	36.01±21.15	34.35		43.37±24.27[df]	35.60	
T3	126 (11.8)	36.52±12.82	34.75		38.62±20.83[fg]	31.31	
T4	29 (2.7)	36.26±12.41	33.54		45.68±21.26[eg]	38.60	
**pN stage** (n=1064)							
N0	578 (54.3)	35.30±11.41	34.97	0.299[a]	41.73±22.17	35.91	0.659[a]
N1	308 (28.9)	37.40±26.83	35.17		40.22±22.07	32.64	
N2	108 (10.2)	35.69±12.11	33.88		40.26±20.70	33.89	
N3	70 (6.6)	34.24±9.72	33.42		39.15±25.42	31.53	
**pM stage**							
M0	1050 (98.1)	35.84±17.37	34.83	0.568[c]	40.82±22.11	34.46	0.091[c]
M1	20 (1.9)	38.07±13.24	38.73		49.28±25.21	39.68	

[a]*p-value were estimated by one-way ANOVA test.*
[b]*p-values were estimated by Kruskal-Wallis 1-way ANOVA test.*
[c]*p-value were estimated by student's T test.*
[d]*p=0.003,* [e]*p=0.018,* [f]*p=0.029,* [g]*p=0.031,*

Supplementary Table S4. Correlation of ALDOA and ENO1 expression with clinicopathological characteristics of breast cancer 1070 patients.

Variables	ALDOA (n=1070)				ENO1 (n=1070)		
	No. (%)	Mean±SD	Median	p-value	Mean±SD	Median	p-value
**Pathology stage**							
I	272 (25.4)	251.04±146.14	210.26	0.100[a]	225.55±144.61	183.02	0.222[b]
II	554 (51.8)	260.70±162.30	215.58		246.47±174.08	182.03	
III	224 (20.9)	286.98±204.28	240.96		222.52±154.43	180.70	
IV	20 (1.9)	281.33±113.31	259.23		327.77±245.09	254.00	
**pT stage**							
T1	355 (33.2)	254.39±153.51	211.57	0.464[a]	223.51±145.24	181.84	0.258[a]
T2	560 (52.3)	266.99±164.43	223.76		244.71±167.70	186.80	
T3	126 (11.8)	271.91±221.36	213.17		242.19±201.07	174.89	
T4	29 (2.7)	294.27±123.45	299.47		254.95±172.15	207.59	
**pN stage** (n=1064)							
N0	578 (54.3)	257.69±160.44	214.66	0.469[a]	250.73±174.87	192.04	0.062[b]
N1	308 (28.9)	267.06±172.14	219.56		216.79±138.75	170.87	
N2	108 (10.2)	283.08±174.05	248.63		234.99±182.89	193.36	
N3	70 (6.6)	273.61±200.68	224.24		228.21±159.73	173.20	
**pM stage**							
M0	1050 (98.1)	263.80±168.63	218.72	0.644[c]	235.94±163.01	182.41	0.062[d]
M1	20 (1.9)	281.33±113.31	259.23		327.77±245.09	254.00	

[a]*p-value were estimated by one-way ANOVA test.*
[b]*p-values were estimated by Kruskal-Wallis 1-way ANOVA test.*
[c]*p-value were estimated by student's T test.*
[d]*p-value were estimated by Mann-Whitney U test.*

Supplementary Table S5. Correlation of GFPT1 and GNPNAT1 expression with clinicopathological characteristics of breast cancer 1070 patients.

Variables	GFPT1 (n=1070)				GNPNAT1 (n=1070)		
	No. (%)	Mean±SD	Median	p-value	Mean±SD	Median	p-value
**Pathology stage**							
I	272 (25.4)	20.02±10.09	16.94	0.902[a]	12.20±5.76	11.14	0.123[b]
II	554 (51.8)	20.76±15.25	17.66		12.60±7.69	10.86	
III	224 (20.9)	20.66±16.41	16.62		14.29±9.86	12.49	
IV	20 (1.9)	19.72±7.74	21.86		12.61±6.60	9.88	
**pT stage**							
T1	355 (33.2)	19.93±9.91	17.05	0.253[a]	12.31±5.99	11.14	0.391[b]
T2	560 (52.3)	21.03±16.02	17.66		13.19±8.80	11.19	
T3	126 (11.8)	19.24±15.87	15.92		12.55±7.54	10.73	
T4	29 (2.7)	24.02±15.83	18.56		14.32±7.37	13.03	
**pN stage** (n=1064)							
N0	578 (54.3)	20.29±13.08	17.31	0.681[a]	12.24±6.56[d]	10.90	0.010[b]
N1	308 (28.9)	21.09±15.15	17.87		13.09±8.05[e]	11.21	
N2	108 (10.2)	21.39±15.16	17.24		15.54±11.40[def]	13.21	
N3	70 (6.6)	19.35±18.22	15.74		12.76±8.27[f]	11.56	
**pM stage**							
M0	1050 (98.1)	20.55±14.36	17.24	0.796[c]	12.86±7.81	11.16	0.888[c]
M1	20 (1.9)	19.72±7.74	21.86		12.61±6.60	9.88	

[a]*p-value were estimated by one-way ANOVA test.*
[b]*p-values were estimated by Kruskal-Wallis 1-way ANOVA test.*
[c]*p-value were estimated by student's T test.*
[d]*p=0.001,* [e]*p=0.028,* [f]*p=0.025,*

Supplementary Table S6. Correlation of PGM3 and UAP1 expression with clinicopathological characteristics of breast cancer 1070 patients.

Variables	PGM3 (n=1070)				UAP1 (n=1070)		
	No. (%)	Mean±SD	Median	p-value	Mean±SD	Median	p-value
**Pathology stage**							
I	272 (25.4)	7.19±2.82	6.69	0.953[b]	38.14±19.32	33.45	0.532[a]
II	554 (51.8)	7.39±4.20	6.56		38.17±18.71	35.28	
III	224 (20.9)	7.38±3.88	6.52		36.08±17.10	31.64	
IV	20 (1.9)	7.30±2.47	6.13		37.60±22.77	25.60	
**pT stage**							
T1	355 (33.2)	7.08±2.81	6.51	0.308[b]	37.22±18.28	33.13	0.132[a]
T2	560 (52.3)	7.53±4.31	6.67		38.77±19.21	35.38	
T3	126 (11.8)	7.05±3.81	6.34		34.62±15.94	31.34	
T4	29 (2.7)	7.78±3.51	7.02		36.83±21.05	30.74	
**pN stage** (n=1064)							
N0	578 (54.3)	7.49±4.10	6.70	0.271[a]	39.46±20.58	35.70	0.052[b]
N1	308 (28.9)	6.99±3.14	6.54		35.09±14.55	32.44	
N2	108 (10.2)	7.54±3.80	6.57		36.92±18.23	32.04	
N3	70 (6.6)	7.36±3.92	6.45		37.49±17.08	31.58	
**pM stage**							
M0	1050 (98.1)	7.33±3.82	6.58	0.970[c]	37.72±18.54	34.13	0.977[c]
M1	20 (1.9)	7.30±2.47	6.13		37.60±22.77	25.60	

[a]*p-value were estimated by one-way ANOVA test.*
[b]*p-values were estimated by Kruskal-Wallis 1-way ANOVA test.*
[c]*p-value were estimated by student's T test.*

Supplementary Table S7. Correlation of PDHB and IDH2 expression with clinicopathological characteristics of breast cancer 1070 patients.

Variables	PDHB (n=1070)				IDH2 (n=1070)		
	No. (%)	Mean±SD	Median	p-value	Mean±SD	Median	p-value
**Pathology stage**							
I	272 (25.4)	14.78±5.27	13.88	0.894[a]	81.50±65.33	65.76	0.198[a]
II	554 (51.8)	14.65±5.54	13.71		86.38±68.51	69.48	
III	224 (20.9)	14.70±5.03	13.79		87.53±64.68	70.59	
IV	20 (1.9)	13.84±5.65	12.52		113.49±89.59	94.04	
**pT stage**							
T1	355 (33.2)	15.07±5.42	14.16	0.228[a]	81.18±62.75	66.59	0.292[a]
T2	560 (52.3)	14.45±5.36	13.46		89.61±71.63	71.33	
T3	126 (11.8)	14.38±5.20	12.99		82.65±64.33	63.99	
T4	29 (2.7)	15.67±5.18	14.22		85.71±47.19	75.89	
**pN stage** (n=1064)							
N0	578 (54.3)	14.49±5.37	13.52	0.254[a]	83.91±61.57	67.15	0.238[a]
N1	308 (28.9)	15.21±5.67	14.19		85.16±73.53	69.31	
N2	108 (10.2)	14.38±5.01	13.74		89.26±68.58	70.39	
N3	70 (6.6)	14.52±4.51	14.43		100.86±83.80	74.00	
**pM stage**							
M0	1050 (98.1)	14.70±5.36	13.75	0.478[b]	85.36±66.87	69.10	0.065[b]
M1	20 (1.9)	13.84±5.65	12.52		113.49±89.59	94.04	

[a]*p-value were estimated by one-way ANOVA test.*
[b]*p-value were estimated by student's T test.*

Supplementary Table S8. Correlation of SDHA and FH expression with clinicopathological characteristics of breast cancer 1070 patients.

Variables	SDHA (n=1070)				FH (n=1070)		
	No. (%)	Mean±SD	Median	p-value	Mean±SD	Median	p-value
**Pathology stage**							
I	272 (25.4)	12.95±6.07[de]	11.94	0.002[a]	44.72±18.79	40.52	0.103[a]
II	554 (51.8)	14.03±5.78	13.09		47.46±20.36	43.50	
III	224 (20.9)	14.47±5.78[d]	13.03		45.10±17.20	42.44	
IV	20 (1.9)	16.82±6.20[e]	16.28		51.71±23.25	46.68	
**pT stage**							
T1	355 (33.2)	13.05±5.63[fgh]	12.06	0.005[b]	44.25±17.85[i]	40.87	0.003[a]
T2	560 (52.3)	14.26±6.05[f]	13.09		48.25±20.40[i]	44.72	
T3	126 (11.8)	14.27±5.42[g]	13.07		43.09±17.82	38.48	
T4	29 (2.7)	15.90±6.72[h]	13.61		49.53±21.60	47.94	
**pN stage** (n=1064)							
N0	578 (54.3)	13.86±6.32	12.57	0.115[b]	46.73±20.51	42.43	0.916[b]
N1	308 (28.9)	13.30±4.58	12.86		46.32±18.54	43.54	
N2	108 (10.2)	14.60±6.05	13.13		45.06±17.49	42.46	
N3	70 (6.6)	15.33±6.18	13.14		45.39±17.44	42.76	
**pM stage**							
M0	1050 (98.1)	13.85±5.87	12.69	0.025[c]	46.25±19.35	42.58	0.213[c]
M1	20 (1.9)	16.82±6.20	16.28		51.71±23.25	46.68	

[a]*p-value were estimated by one-way ANOVA test.*
[b]*p-values were estimated by Kruskal-Wallis 1-way ANOVA test.*
[c]*p-value were estimated by student's T test.*
[d]*p=0.043,* [e]*p=0.044,* [f]*p=0.004,* [g]*p=0.020,* [h]*p=0.017,* [i]*p=0.026*

Supplementary Table S9. Univariate and multivariate Cox's regression analysis of gene expression for overall survival of 1070 patients with breast cancer.

Characteristic	No. (%)	OS			
		CHR (95% CI)	P-value	AHR (95% CI)	P-value
**HK1**	(n=1070)				
Low	1017 (95.0)	1.00		1.00	
High	53 (5.0)	2.80 (1.34-5.84)	0.006	2.18 (1.04-4.59)	0.040
**GPI**					
Low	715 (66.8)	1.00		1.00	
High	355 (33.2)	1.55 (0.97-2.49)	0.067	1.71 (1.05-2.77)	0.030
**ALDOA**					
Low	818 (76.4)	1.00		1.00	
High	252 (23.6)	1.44 (0.85-2.43)	0.178	1.44 (0.85-2.45)	0.174
**ENO1**					
Low	772 (67.5)	1.00		1.00	
High	348 (32.5)	2.21 (1.39-3.52)	0.001	2.15 (1.33-3.46)	0.002
**GFPT1**					
Low	764 (71.4)	1.00		1.00	
High	306 (28.6)	1.34 (0.82-2.20)	0.239	1.31 (0.79-2.17)	0.288
**GNPNAT1**					
Low	1016 (95.0)	1.00		1.00	
High	54 (5.0)	2.17 (0.99-4.73)	0.052	1.53 (0.69-3.38)	0.294
**PGM3**					
Low	943 (88.1)	1.00		1.00	
High	127 (11.9)	1.79 (0.98-3.27)	0.057	1.61 (0.88-2.95)	0.122
**UAP1**					
Low	220 (20.6)	1.00		1.00	
High	850 (79.4)	1.44 (0.74-2.81)	0.286	1.49 (0.76-2.91)	0.244
**PDHB**					
Low	462 (43.2)	1.00		1.00	
High	608 (56.8)	0.56 (0.35-0.89)	0.015	0.60 (0.38-0.96)	0.034
**IDH2**					
Low	555 (51.9)	1.00		1.00	
High	515 (48.1)	1.70 (1.05-2.73)	0.029	1.55 (0.96-2.50)	0.075
**SDHA**					
Low	693 (64.8)	1.00		1.00	
High	377 (35.2)	2.19 (1.37-3.48)	0.001	1.93 (1.21-3.09)	0.006
**FH**					
Low	682 (63.7)	1.00		1.00	
High	388 (36.3)	1.37 (0.86-2.19)	0.189	1.26 (0.78-2.03)	0.338

Abbreviation: DSS, disease-specific survival; DFS, disease-free survival; CHR, crude hazard ratio; AHR, adjusted hazard ratio

AHR were adjusted for AJCC pathological stage (II,III and IV VS. I).
